# Studying trabecular bone samples demonstrates a power law relation between deteriorated structure and mechanical properties - a study combining 3D printing with the finite element method

**DOI:** 10.3389/fendo.2023.1061758

**Published:** 2023-06-02

**Authors:** Xiuhong Huang, Liqin Zheng, Desheng Zheng, Shaobin Li, Yueguang Fan, Ziling Lin, Shaohong Huang

**Affiliations:** ^1^ Stomatological Hospital, School of Stomatology, Southern Medical University, Guangzhou, China; ^2^ The First Clinical Medical College, Guangzhou University of Chinese Medicine, Guangzhou, Guangdong, China; ^3^ Department of Joint Surgery, The First Affiliated Hospital of Guangzhou University of Chinese Medicine, Guangzhou, China; ^4^ Department of Orthopedic Trauma, The First Affiliated Hospital of Guangzhou University of Chinese Medicine, Guangzhou, Guangdong, China

**Keywords:** osteoporosis, trabecular bone, biomechanics, 3D printing, finite element method, side-artifact

## Abstract

**Introduction:**

The bone volume fraction (BV/TV) significantly contributes to the mechanical properties of trabecular bone. However, when studies compare normal trabeculae against osteoporotic trabeculae (in terms of BV/TV decrease), only an “average” mechanical result has been determined because of the limitation that no two trabecular structures are the same and that each unique trabecular structure can be mechanically tested only once. The mathematic relation between individual structural deterioration and mechanical properties during aging or the osteoporosis process has yet to be further clarified. Three-dimensional (3D) printing and micro-CT-based finite element method (μFEM) can assist in overcoming this issue.

**Methods:**

In this study, we 3D printed structural-identical but BV/TV value-attenuated trabecular bones (scaled up ×20) from the distal femur of healthy and ovariectomized rats and performed compression mechanical tests. Corresponding μFEM models were also established for simulations. The tissue modulus and strength of 3D printed trabecular bones as well as the effective tissue modulus (denoted as Ez) derived from μFEM models were finally corrected by the side-artifact correction factor.

**Results:**

The results showed that the tissue modulus _corrected_, strength _corrected_ and Ez _corrected_ exhibited a significant power law function of BV/TV in structural-identical but BV/TV value-attenuated trabecular samples.

**Discussion:**

Using 3D printed bones, this study confirms the long-known relationship measured in trabecular tissue with varying volume fractions. In the future, 3D printing may help us attain better bone strength evaluations and even personal fracture risk assessments for patients who suffer from osteoporosis.

## Introduction

Osteoporosis is characterized by the impairment of bone mass, strength, and microarchitecture, which increases the propensity of fragility fractures (1). The mechanical properties of trabecular bone are mainly determined by the architectural arrangement of the network. Typically, the calculated trabecular 3D microstructure includes bone volume fraction (BV/TV), trabecular number (Tb. N), trabecular thickness (Tb. Th), trabecular spacing (Tb. Sp), connectivity density (Conn. D), structure model index (SMI) and the degree of anisotropy (DA) (2). Although trabecular networks are estimated by these different structural parameters, BV/TV is the main determinant of trabecular bone mechanical properties. An estimated 70-80% of the variances in the apparent tissue modulus of trabecular bone can be explained by BV/TV (3–6). The apparent modulus and collapse stress of regular foams (simulating trabecular tissue) are predicted to be quadratic functions of BV/TV (7), especially when accounting for the side-artifacts of cored trabecular specimens (8, 9). There are different regression relations between BV/TV and the tissue modulus of fractured and nonfractured proximal femurs, indicating that the spatial arrangement and attenuation of trabeculae could jeopardize the mechanical properties of trabecular bone (10, 11).

Trabecular samples tested in these studies were obtained from a set of specimens that were pooled from multiple individuals collectively spanning a wide range of densities and BV/TV; when tissue modulus and BV/TV were correlated regardless of side-artifact correction, the goodness of fit was better with the addition of other structural indices, e.g., DA (4), Tb. Sp and mean intercept length (MIL) (3). Since no two trabecular structures are exactly the same and osteoporosis is not necessarily systemic (12), it is almost impossible to control the specific architecture of each trabecular specimen and to correlate their structure to its function. Furthermore, even with similar BV/TV values, healthy and osteoporotic trabeculae may yield significantly different mechanical properties (13). To date, the deterioration of trabecular structure (e.g., from a healthy state to osteoporosis) and the resulting changes in mechanical properties in individuals remain to be better clarified.

The finite element method based on micro-CT (μFEM) has been widely used to study trabecular bone mechanics (14–17). The ability to test each trabecular sample multiple times is greatly beneficial, either along with different loading modes or from different loading directions. For example, Maquer (4) analyzed 743 micro-CT reconstructions of cubic trabecular samples using μFEM to confirm the significant role of both BV/TV and DA in determining apparent elastic properties. Using μFEM, Liu (18) loaded the same trabecular cubes from three principal directions (along the X, Y and Z axes) to suggest that (1) BV/TV alone has a significant effect on the tissue modulus of the trabecular bone, while tissue properties demonstrated little effect on it; and (2) under the same BV/TV value, microarchitecture has superior mechanical properties along the principal direction of loading. Through μFEM, the stress and strain within trabeculae under loading can be estimated, as well as bone formation/resorption (19). However, μFEM simulation needs to be validated with *in vitro* mechanical testing to confirm that accurate mechanical properties are predicted. This requirement is impossible to attain in μFEM simulations that manipulate an existing trabecular structure, such as bone remodeling or osteoporosis, as the derived trabecular structures do not physically exist (20).

Recently, 3D printed (3DP) trabecular bone has been used to demonstrate the effect of structure deterioration on stiffness and strength. With the help of a high-resolution 3D printer, the 3DP trabecular sample is highly consistent with the actual trabecular structure scanned by micro-CT (21). A decrease of approximately 8% was calculated in the BV/TV of 3DP trabecular bones yielded a corresponding reduction in structural stiffness (17%) and strength (24%), which agrees with *in vitro* studies that were performed using actual trabecular samples (20). Although there is little information about the true values of strength and stiffness of actual trabecular bone, the decline rate of 3DP trabecular geometries still effectively explains part of the structure−function relationship.

Because μFEM and 3D printing show advantages in replicating the three-dimensional microstructure of trabeculae and reflecting the mechanical behavior, the overall goal of this study is to establish a validated method of given 3DP trabecular geometries simulating bone loss during osteoporosis to evaluate the mechanical properties and simulate them using μFEM, accounting for side-artifact correction. Specifically, our objectives are to (1) 3D print a cohort of trabecular samples (scaled up 20 times, from a rat’s distal femur) sharing the same underlying architecture but with a series of different BV/TV. This was achieved by changing the global threshold during the segmentation process. (2) Then, the 3DP trabecular samples were tested in compression until failure to calculate the tissue modulus and strength. (3) Next, μFEM models of the segmented trabecular samples (1:1 size) were developed, and the effective tissue modulus was calculated by dividing the applied stress by the resultant strain. (4) Finally, the BV/TV values were correlated to the corrected mechanical properties (corrected by side-artifact correction factor) acquired from compressive tests and μFEM simulation. This study is novel in that we combine 3D printing and uFEM to quantify the relation between the mechanical properties and the given continuously deteriorated trabecular bone while taking side artifacts into account.

## Materials and methods

### Animals and samples

The animal research protocol was approved by the ethics committee of Guangzhou University of Chinese Medicine. A total of 6 2-month-old female Sprague−Dawley (SD) rats were randomly separated into a normal group (N, n=3) and an ovariectomized group (OVX, n=3). All rats were raised in a standard specific-pathogen-free (SPF) environment and allowed free access to food. After 12 weeks, all rats (N, n=3; OVX, n=3) were euthanized, and the right femurs were dissected and collected. Soft tissues connected to the femur were removed, and the femur was fixed with 4% paraformaldehyde and stored at room temperature (25 °C) for micro-CT scanning.

### Micro-CT scanning

**Figure 1:**
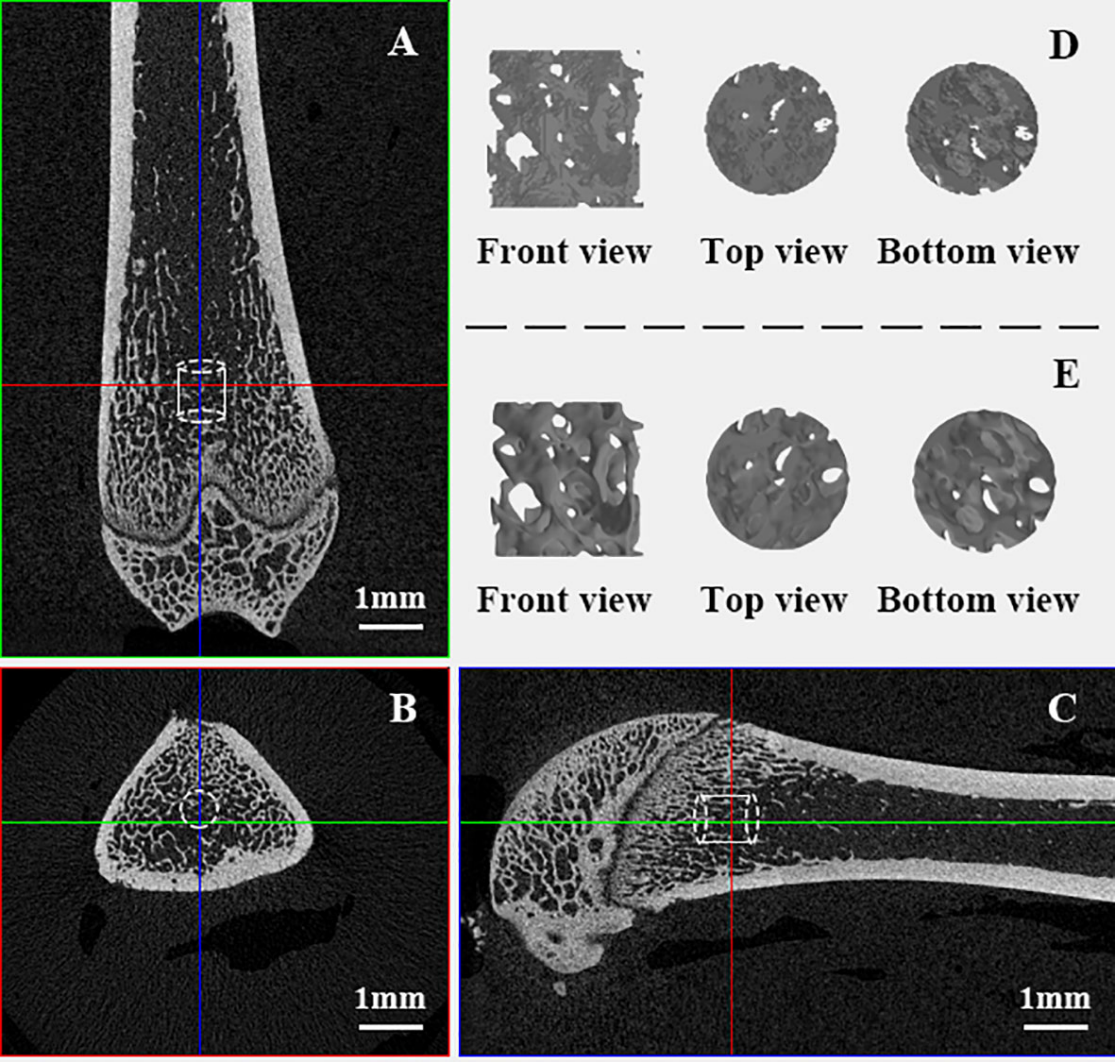
VOI trabeculae in distal femoral metaphysis. **(A–C)**: VOI taken from the domain of trabeculae in femur samples; **(D)**: Raw “stl” file format VOI trabeculae cylinder full of noises; **(E)**. VOI trabeculae cylinders after optimization.

The excised right femurs were scanned using a high-resolution micro-CT scanner (SkyScan1172, Bruker, USA) at a resolution of 15 μm, with a voltage of 80 kV, a current of 100 μA, a 0.5 mm aluminum filter, a rotation step of 0.6° and an exposure time of 360 ms. Images were then reconstructed using bundled software Nrecon1.7. Another bundled software, DataViewer1.4.3, was employed to orient the cross-sectional images parallel to the transaxial plane. A cylindrical volume of interest (VOI) with a diameter of 1 mm and height of 1 mm was placed randomly in the distal femoral metaphysis. See an example shown in **Figures 1A–C**.

**Figure 2:**
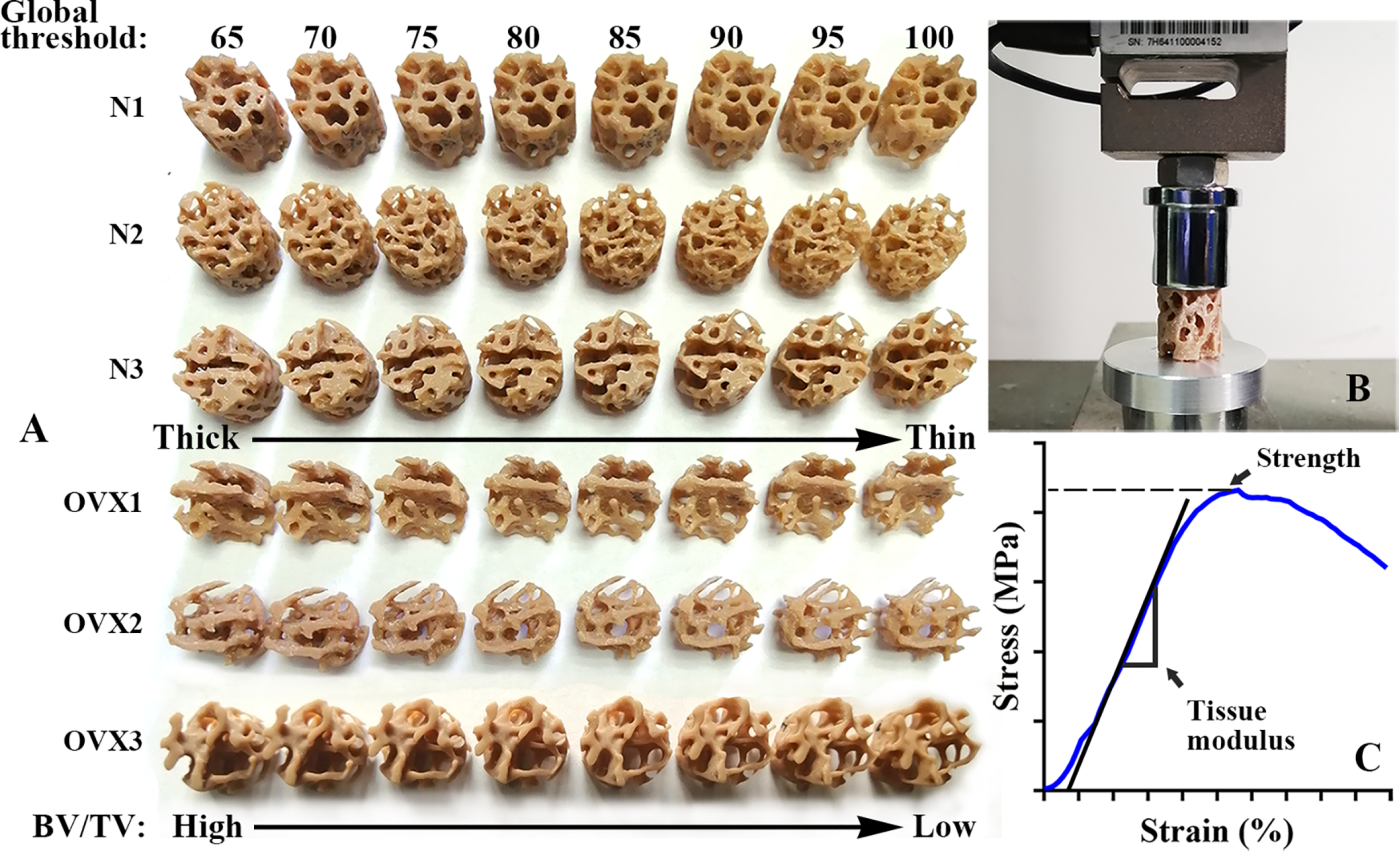
3DP VOI trabeculae and mechanical test. **(A)** An overview of 3DP VOI trabeculae from N group and OVX group. A cohort of global thresholds lower limit from 65 to 100 at an interval of 5 was chosen to segment out trabeculae under the same VOI region. With the global threshold increase, the BV/TV value decrease and the 3DP trabeculae structure deteriorate. **(B)** A view of universal testing machine used to load the 3DP VOI trabeculae in compression. **(C)** A typical stress–strain curve of 3DP VOI trabeculae tested along the principal direction.

Morphometric analysis of the VOI trabecular bone was performed using CTAn1.16 (SkyScan1172, Bruker, USA). A cohort of global threshold lower limits ranging from 65 to 100 at an interval of 5 was chosen to segment out trabeculae under the same VOI region. Different global thresholds will lead to microstructural models exhibiting various degrees of osteoporosis. The higher the global threshold chosen, the higher the degree of simulated osteoporosis (**Figure 2A**). The trabecular parameters BV/TV, Tb. Th, Tb. Sp, were measured for each VOI. The VOI trabecular bones were also saved in “stl” file format for subsequent 3D model optimization and 3D printing.

### 3D model optimization

The raw segmentations derived from CTAn software were noisy (for example, full of spikes and unconnected elements, as **Supplementary Figure S1** shows). They were too rough for 3D printing and finite element analysis (**Figure 1D**), and some smoothing steps were undertaken prior to printing and volume meshing. In Geomagic studio 2013 software (Geomagic, United States), the “Mesh Doctor” option was used to automatically detect and repair defects, and then the “Remove Spikes” function was used (the smoothing level was selected as 10 (the level is 0-100) to remove small spikes, but the microstructure characteristics of the trabeculae were retained as much as possible), and Remesh was used to obtain 2D elements with uniform distribution(the element length was set to 8 μm, which is much smaller than rats’ actual trabeculae sizes of 15-194 μm in our samples). The structural difference between the postsmoothing VOI trabecular sample and the original file can be found in **Supplementary Figure S2**. These postsmoothing 3D models were saved in “stl” file format again, ready for subsequent 3D printing and μFEM preprocessing (**Figure 1E**).

### 3D printing

3D printing can accurately and precisely replicate trabecular specimens and produce informative mechanical alterations (20). The actual VOI trabecular structure (1 mm in diameter, 1 mm in height) was too small to be fabricated and replicated. Therefore, before printing, the VOI trabecular bone was scaled up 20 times (22) using Materialise Magics21.0 software (Materialise, Belgium). The poly lactic acid (PLA) filament with a layer thickness of 80 μm was selected to use in an HP jet fusion 3D printer (Hewlett Packard, USA). The layer thickness is approximately 3.75-48.5 times the size of magnified VOI trabeculae (**Figure 2A**). The HP Jet Fusion 3D printer uses a novel technique called Multi-Jet Fusion (MJF), which offers low machining time, competitive part properties and minimal postproduction finishing compared to existing 3D printing technologies (23, 24). PLA is an inexpensive and readily available material. According to the manufacturer, PLA filaments have a melting temperature of 170-230°C and a density of approximately 1.35 g/cm³. Before testing 3DP trabecular samples, it is necessary to confirm that the 3D printer can accurately copy the microstructure. This verification can be carried out by regression between the weight of the 3DP trabecular sample and their BV/TV value since all VOI trabecular bone was printed by the same filament. The 3DP trabecular bones were weighed three times to obtain the average weight, and the least square linear regression was developed according to BV/TV to verify the accuracy of the replicated microstructure (20).

### Mechanical tests

3DP trabecular bones were uniaxially compressed using a universal testing machine equipped with a 10 kN load cell (Jingzhuo Machinery Factory, Yangzhou, China), with an error in the indicating value of 0.3%. 3DP trabecular bones were loaded at a rate of 2 mm/min (i.e., strain rate of 0.1/min). A small compression preload of 5 N was applied at the beginning of each experiment (**Figure 2B**). The measurements were recorded at 10 Hz (every 100 milliseconds). The tissue modulus and strength were determined where the tissue modulus was defined as the slope of the stress−strain curve in the linear region and the strength was defined as the peak of the stress−strain curve (**Figure 2C**).

### Finite element analysis

**Figure 3:**
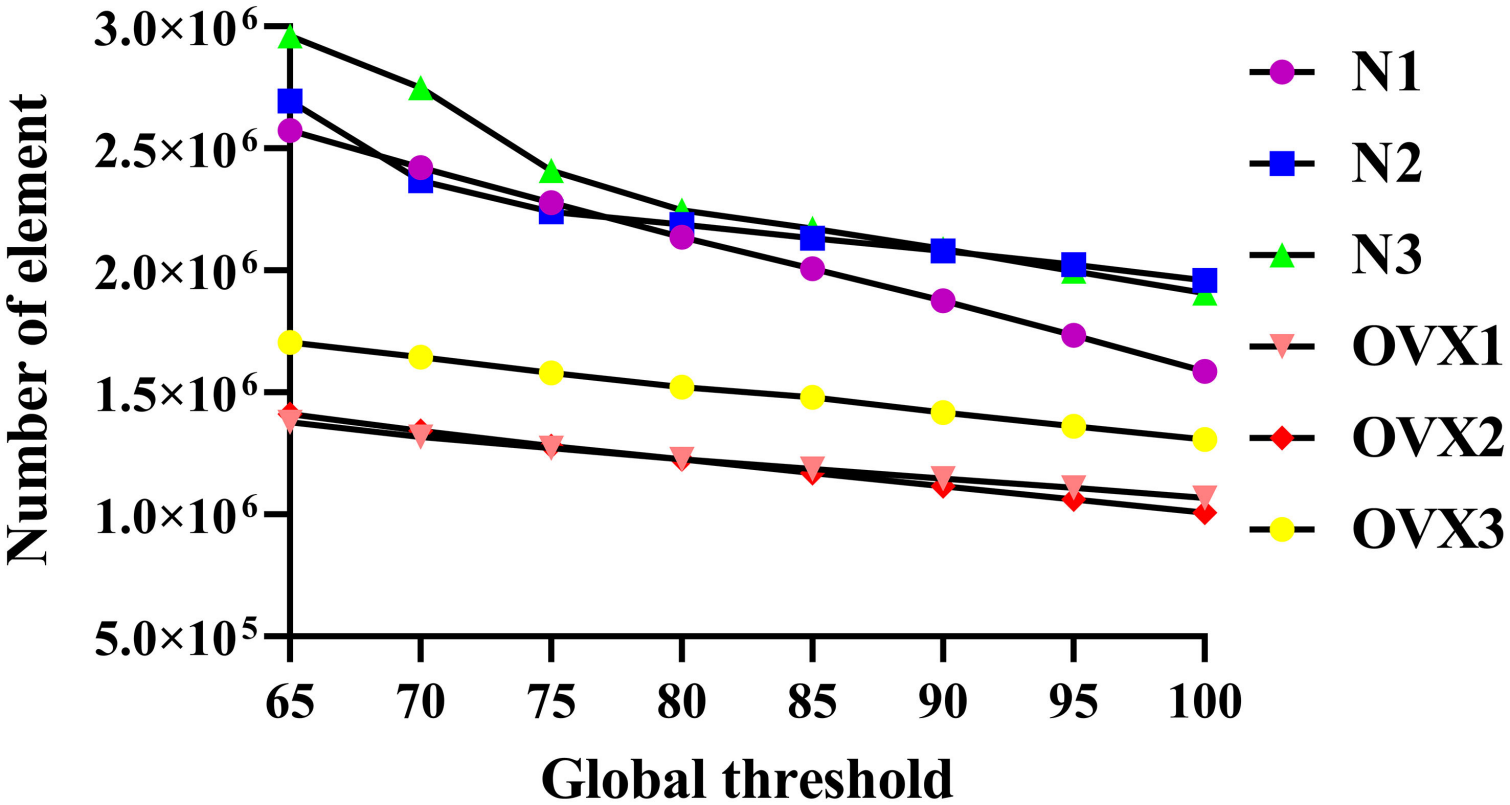
Number of μFEM models’ element.

The μFEM was developed to obtain the effective tissue modulus (denoted as Ez) of VOI trabecular bone (18). The postoptimized VOI trabecular bones were meshed to four-node tetrahedral elements (C3D4) using Hypermesh 14.0 software (Altair, USA), with an average size of 8 μm, which is smaller than the rats’ actual trabecular dimensions so that the details of the 3D architectures were well maintained. Our sensitivity analysis suggests that 8 μm is sufficient to converge (**Supplementary Figure S3**). As the global threshold increases, the number of μFEM model elements decreases (**Figure 3**).

The μFEM model was considered linear elastic with a Poisson’s ratio of 0.3 (25). The Young’s modulus of the VOI trabecular bone was assigned according to the relation derived from the rat’s distal femoral trabeculae (26):


$$\text{E}=14899{\left(\text{BV}/\text{TV}\right)}^{1.94}$$

where E represents Young’s modulus. In this study, we attempted to investigate the effect of microarchitecture deterioration on the mechanical properties of trabecular tissue. Therefore, the modulus assigned to the μFEM trabeculae element will be consistent within each group. The average BV/TV values of the N and OVX groups were 42.19% and 25.25%, respectively, and their Young’s moduli were approximately 3000 MPa and 900 MPa, respectively.

**Figure 4:**
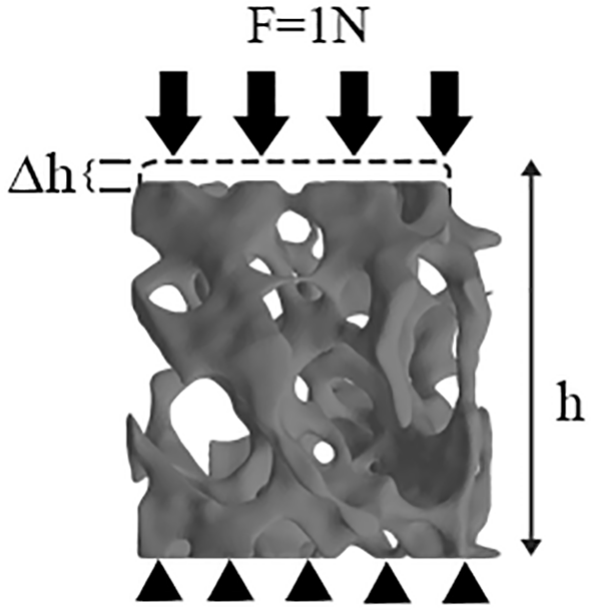
μFEM model of VOI trabeculae. Black arrows represent load by means of loading control, black triangles represent boundary condition.


A small uniaxial compressive force (F=1 N) (27) was applied along the Z axis (i.e., the principal direction), and the resultant displacement was therefore computed (**Figure 4**). We denoted r, h, and Δh as the radius, original height, and maximal displacement of VOI trabecular bone, respectively, and F as the applied force. The stress was acquired by F/πr², and the strain by Δh/h. Specifically, the cutoff values for the upper 95th percentile of those nodal displacements (the top 5% of nodal displacement) along the Z axis in each VOI trabecular bone were defined to represent the maximal displacement (28, 29). The maximal displacement was calculated by averaging the top 5% of nodal displacements. Therefore, the Ez of VOI trabecular bone can be derived by the following equation:

$$\text{Ez=}\frac{\text{F}}{{\text{πr}}^{2}}/\frac{\text{Δh}}{\text{h}}$$

With this approach, Ez was obtained.

### Side-artifact correction

The cored bone specimen can be considered unloaded near its sides, resulting in errors in the calculated tissue modulus and strength compared to the in situ values in which all trabeculae would carry load. This experimental artifact is called ‘side-artifact’ (8, 9). Due to trabeculae on the sample border losing connectivity and therefore load-bearing capacity, the true modulus and strength need to be corrected by a side-artifact correction factor. The correction factor tends to markedly increase as BV/TV decreases, especially when using a small trabecular sample (8). Both 3DP and computational models in this study will suffer from side-artifacts. To assess the radial distribution of stresses for identification of the region affected by the side-artifact, we examined von-mise stresses on radial rings of thickness 0.05 mm on 10 equally spaced transverse cross sections for each trabecular model. The presence of the side-artifact was characterized by a statistically significant reduction in stresses for the outer radial rings relative to the inner radial rings. The corresponding thickness (t) of outer radial rings was used to calculate the side-artifacts correction factor according the following equation (8):


$$Correction\;factor={\left(\frac{\text{D}}{\text{D}-2\text{t}}\right)}^{2}$$

Where D is the diameter of trabecular sample. BV/TV value and correction factor of each sample can be found in **Supplementary Table S1**. The corrected tissue modulus, strength and Ez were respectively denoted as tissue modulus _corrected_, strength _corrected_ and Ez _corrected_.

### Statistical analysis

A previous study theoretically predicted that the tissue modulus and strength of isotropic foams (similar to trabecular bone regardless of anisotropic mineral substance and collagen distribution) are quadratic functions of BV/TV (7). Given the 3DP trabecular bones corrected by the side-artifact correction factor, in the present study, a nonlinear regression function (SPSS20.0, IBM, USA) was established to determine the effect of deteriorated BV/TV in the form:


$$Y=A\cdot {\left(BV/TV\right)}^{\wedge }B,$$

where *Y* is one of three measured dependent variables (i.e., tissue modulus _corrected_, strength _corrected_ and Ez _corrected_), *A* and *B* are the derived coefficients.

## Result

### 3D printing accuracy validation

**Figure 5:**
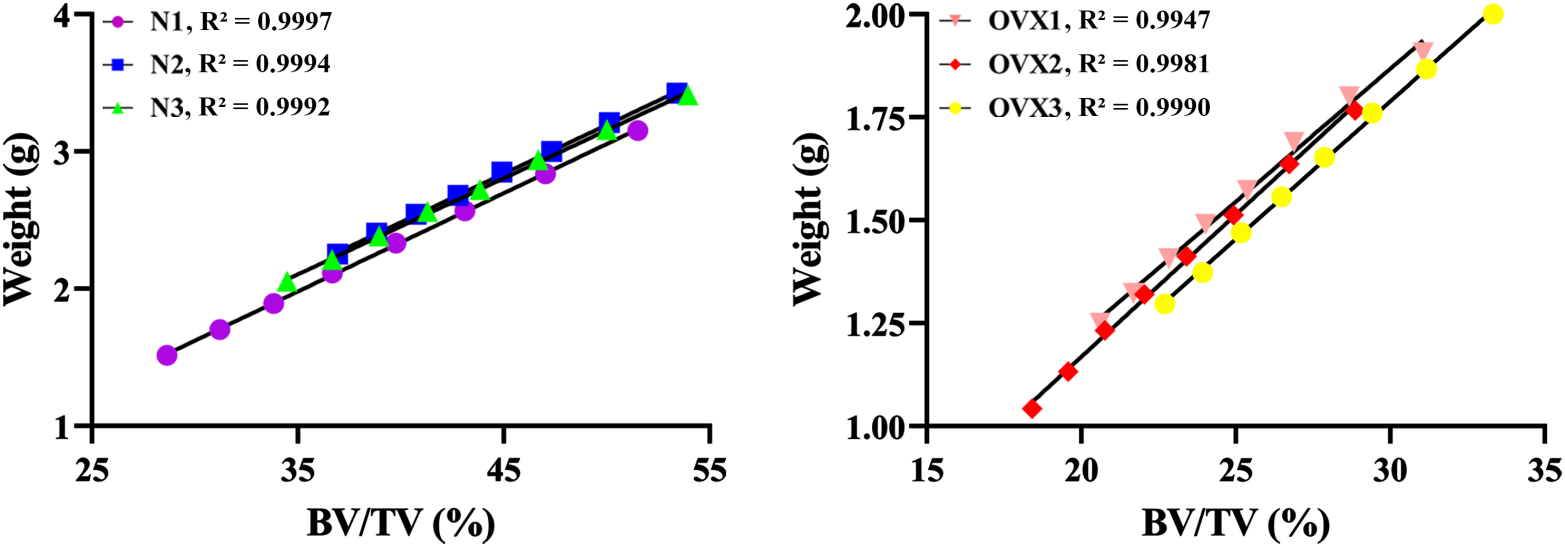
3DP accuracy validation. Linear regression between BV/TV and the weight of 3DP VOI trabeculae.

**Figure 5** shows that with BV/TV increase, N1 weight increased from 1.517 g to 3.153 g, N2 weight increased from 2.253 g to 3.427 g, N3 weight increased from 2.057 g to 3.413 g; OVX1 weight increased from 1.250 g to 1.907 g, OVX2 weight increased from 1.043 g to 1.767 g, and OVX3 weight increased from 1.297 to 2.000 g. The weight of 3DP trabecular bones was found to significantly correlate with their BV/TV in both the N and OVX groups (R² > 0.99). It is therefore reasonable to assume that the 3DP trabecular bones replicated the actual microstructure well, and the 3D printer we used can accurately print the trabecular model.

### Structural deterioration

**Figure 6:**
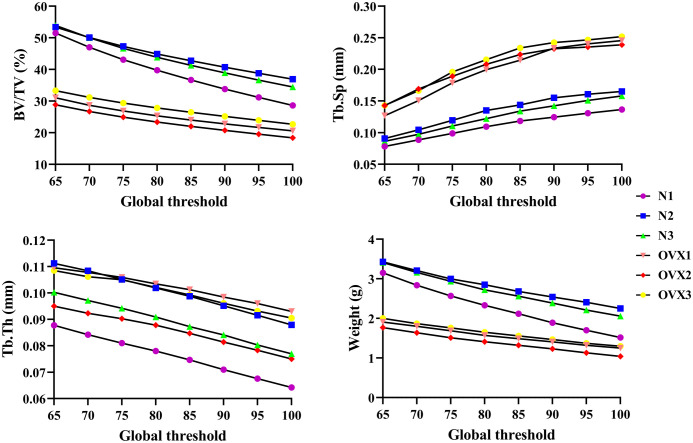
Structural parameter and the weight of VOI trabeculae at different global threshold.

As **Figure 6** shows, as the global threshold limit increased from 65 to 100, the BV/TV of N1, N2, and N3 VOI trabecular bone decreased from 51.51%, 53.41%, and 53.94% to 28.64%, 36.92%, and 34.47%, respectively; the BV/TV of OVX1, OVX2, and OVX3 VOI trabecular bone decreased from 31.06%, 28.86%, and 33.33% to 20.61%, 18.40%, and 22.70%, respectively. Similarly, Tb. Th of N1, N2, and N3 decreased from 0.0877 mm, 0.1112 mm, and 0.1003 mm to 0.0642 mm, 0.0879 mm, and 0.0770 mm, respectively. Tb. Th of OVX1, OVX2, and OVX3 decreased from 0.1096 mm, 0.0950 mm, 0.1085 mm to 0.0930 mm, 0.0750 mm, 0.0905 mm, respectively. Tb. Sp of N1, N2, and N3 increased from 0.0782 mm, 0.0908 mm, and 0.0864 mm to 0.1366 mm, 0.1651 mm, and 0.1580 mm, respectively. Tb. Sp of OVX1, OVX2, and OVX3 increased from 0.1268 mm, 0.1428 mm, and 0.1429 mm to 0.2455 mm, 0.2388 mm, and 0.2517 mm, respectively. The weight of 3DP trabecular bones demonstrated decreasing trends similar to those of BV/TV. OVX trabecular samples show lower BV/TV and corresponding weight.

### Nonlinear regression

**Figure 7:**
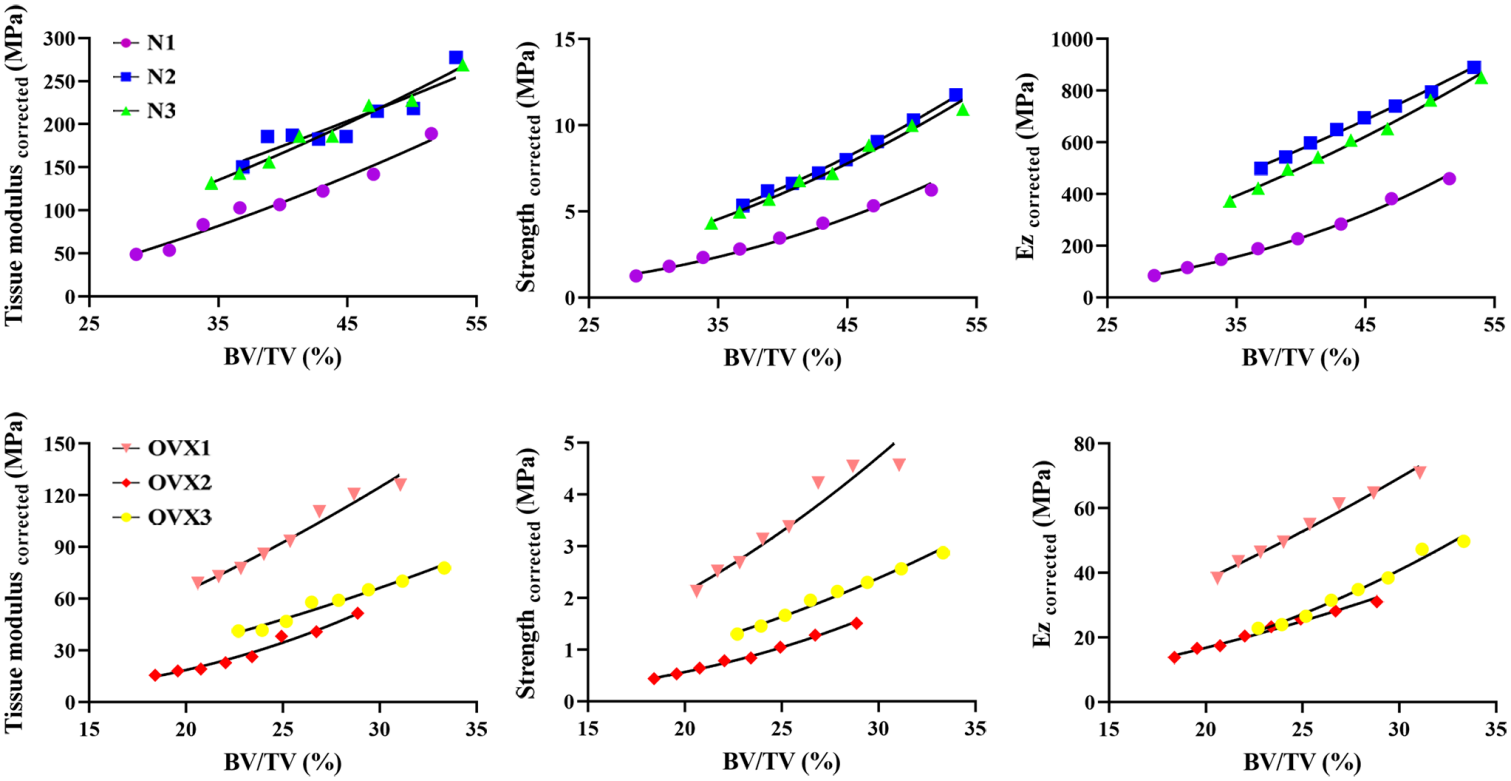
Nonlinear regression for tissue modulus _corrected_, strength _corrected_ and Ez _corrected_ as a function of BV/TV.

As **Figure 7** demonstrates, in the N group, deteriorated BV/TV was found to significantly correlate with tissue modulus _corrected_ as well as strength _corrected_ and Ez _corrected_ in a manner of power law. This significant correlation was also found in the OVX group. Specifically, the normal specimens display a steeper slope than OVX. The detailed coefficients, R² and P value of the nonlinear regression can be found in **Tables 1**–**3**.

**Table 1:** Coefficient of nonlinear regression *(Y = A · (BV/TV)^B)* for tissue modulus.

Sample	A	B	R²	P value
N1	0.026	2.309	0.949	<0.001
N2	2.864	1.175	0.836	=0.001
N3	0.752	1.520	0.976	<0.001
OVX1	0.294	1.843	0.982	<0.001
OVX2	0.003	2.970	0.978	<0.001
OVX3	0.097	1.985	0.972	<0.001

**Table 2:** Coefficient of nonlinear regression *(Y = A · (BV/TV)^B)* for strength.

Sample	A	B	R²	P value
N1	1.590×10^-4^	2.758	0.982	<0.001
N2	5.636×10^-3^	1.968	0.996	<0.001
N3	3.479×10^-3^	2.080	0.986	<0.001
OVX1	3.214×10^-3^	2.210	0.961	<0.001
OVX2	1.080×10^-4^	2.937	0.995	<0.001
OVX3	1.246×10^-3^	2.289	0.989	<0.001

**Table 3:** Coefficient of nonlinear regression *(Y = A · (BV/TV)^B)* for Ez.

Sample	A	B	R²	P value
N1	0.005	2.954	0.994	<0.001
N2	4.035	1.405	0.992	<0.001
N3	0.956	1.757	0.988	<0.001
OVX1	0.258	1.710	0.991	<0.001
OVX2	0.054	1.997	0.988	<0.001
OVX3	0.013	2.436	0.987	<0.001

## Discussion

In this study, the deterioration of trabecular structure in normal and ovariectomized samples was characterized by increasing the global threshold of micro-CT image segmentation, and the mechanical properties of 3DP VOI trabecular bones were quantified after 3DP accuracy validation. Finally, the μFEM was employed to calculate the effective modulus. The results showed that (1) a series of global thresholds chosen to segment out VOI trabecular bone effectively varied the BV/TV value; (2) 3D printing was able to accurately replicate the trabecular structure and provide high-quality 3DP trabecular specimens; (3) a uniaxial compression test on 3DP trabecular bones demonstrated that deteriorated BV/TV was significantly correlated with tissue modulus _corrected_ and strength _corrected_; and (4) μFEM models well reproduced the trend between BV/TV and Ez _corrected_. These results suggest that within an individual deteriorated trabecular bone, tissue modulus and strength are determined by BV/TV in a manner of power law.

Studies involving structural differentiation between healthy and osteoporotic trabecular samples have shown that the BV/TV of osteoporotic individuals decreases by 10%-60%, depending on the anatomical site, sex and age (20, 30). The method to change the global threshold of scanned images to simulate individual bone resorption and transfer into μFEM for mechanical study has been proposed (31). In our research, through a series of global thresholds applied to micro-CT scanned images, the BV/TV value maximally decreased by 28.64%, 16.50%, 19.46%, 13.46%, 10.46% and 9.75% in the N1, N2, N3, OVX1, OVX2 and OVX3 samples, respectively. These decrements support our attempt to segment out the VOI trabeculae characterized by continuous deterioration.

Previous studies have quantified the effect of BV/TV decrease on mechanical properties. For example, a reduction of 8-10.4% in BV/TV led to a 13-17% decrease in stiffness (31, 32), and the ability of BV/TV to explain the variance of elastic properties was more than 70% (3, 26, 33). However, as mentioned above, no two trabecular structures are the same. When these studies compare normal trabeculae against osteoporotic trabeculae (in terms of BV/TV decrease), they only reveal an “average” mechanical result. It is impossible to quantify the mechanical effect of individual structural deterioration for each actual bone sample simply because we cannot test the same sample in various states (20), not to mention the dynamic deterioration process. 3D printing provides a solution to overcome this issue since the correlation coefficient (R²) of the mechanical properties between 3DP and actual trabecular bone samples can up to 0.94 (34).

In actual bone samples, tissue modulus and strength appear to be linear within a single anatomic site but to be power-law across multiple sites because of the differentiation of apparent density (ρ in g/cm^3^) and BV/TV (30, 35). However, these in vitro tested trabecular specimens inevitably suffered from the side-artifacts effect, which results from interruption of the trabecular network along the sides of segmented specimens. Side artifacts are the underestimation of the true mechanical properties, particularly when testing small-size and low-density trabecular bone. Side-artifact correction is essential for obtaining accurate mean estimates of mechanical properties for a cohort of specimens.

The modulus and apparent collapse stress (strength) of an intact open-cell foam model (7) were calculated to be quadratic functions of the volume fraction. Given the side-artifact correction factor, the segmented trabecular specimen can be thought of as an in vitro intact foam, and therefore, its mechanical properties should conform to a quadratic function of BV/TV. However, the tissue modulus _corrected_, strength _corrected_ as well as Ez _corrected_ did not precisely demonstrate a quadratic relation as a function of BV/TV (for example, the exponents B varied between 1.405 and 2.954 in μFEM models). This is because the trabecular tissue is not simply an assembly of regular trabeculae, but a functionalized pore structure with varying trabecular orientation, thickness, length and shape (“rod-like”, “plate-like” and their composite) (4, 33, 36). In fact, the exponents B were not exactly equal, but varied around 2, according to the majority of previous studies involving mechanical tests (30, 35–38). According to the cellular solid theory, exponents B were interpreted as indicators of dominant deformation mechanisms within the trabecular structure. For example, a predominance of axial deformation would lead to a linear relationship, whereas a quadratic relationship would imply that cell wall/strut bending dominates (35, 39). The clustering of exponents for our 3DP models in the range of 1.175–2.970 might therefore be interpreted to suggest that deformation mechanisms are similar across normal and osteoporotic state and involve appreciable bending. The slope of the function in this study is an indicator of geometry attenuation, which suggests that normal trabecular bone is more susceptible to a decrease in bone fraction. This tendency can be verified when we refer to real bone tissue, where bone tissue with a higher bone fraction shows a steeper slope than bone tissue with a lower bone fraction (26).

Few previous studies have used a 3DP trabecular model to simulate the actual trabecular bone (21, 40–43) and test its mechanical properties (20, 44, 45); however, these studies mainly focused on the printing accuracy rather than the structure−function relation, as we did in our study. Dobson et al. (46) used stereolithography 3DP models to validate FE predictions for trabecular bone structures. The 3DP models were tested in compression, and their stiffness values demonstrated a strong correlation with the predictions by the FE analysis. The authors concluded that 3DP models are an important technique to complement the use of FE models for the assessment of the mechanical properties of complex trabecular bone structures. 3DP models are the only way to validate FE models of trabecular bone remodeling, as the derived structures do not physically exist. In this study, we compared the same trabecular structure with a series of BV/TV value reductions in several trabecular samples and established their structure−function relation, which distinguishes ours from previous studies.

This study confirms the long-known relationship measured in trabecular tissue with different volume fractions using 3D printing, suggesting promising applications in skeletal biomechanics (45, 47, 48). However, several potential limitations in our study should be noted. First, we cannot claim that changing the global threshold actually simulates the complex biological process of osteoporosis. In particular, even/global structure deterioration could result in misinterpretations of the influence of trabeculae loss on biomechanical behavior. Simulated bone deterioration offers an opportunity or a tool to investigate some phenomenological aspects of bone loss (49, 50). Second, 3DP filaments are isotropic, while actual bone tissue is hierarchical and anisotropic. This may to some extent reduce the biological significance of our results. Third, 3D-printed trabecular bone may exhibit anisotropy due to the inherently layered construction (e.g., print along the X axis, Y axis and Z axis), but our printed samples were tested orthogonally in the direction of printing. This possible caveat should not affect our results. Last but not least, a broader range of thresholds simulating severe osteoporosis and more parallel trabecular samples (replicated by 3DP) are helpful to prove the structure−function rule more accurately.

## Data availability statement

The original contributions presented in the study are included in the article/**Supplementary Material**. Further inquiries can be directed to the corresponding authors.

## Ethics statement

The animal study was reviewed and approved by Laboratory Animal Ethics Committee of Guangzhou University of Chinese Medicine.

## Author contributions

XH and LZ designed the overall study workflow, analyzed the data, prepared the figures and tables, authored and reviewed drafts of the manuscript, and approved the final draft. DZ and SL aided in analyzing the data, prepared the figures and tables during the revision process. YF, ZL and SH devised the main conceptual idea, supervised the whole work, and approved the final draft. All authors contributed to the article and approved the submitted version.

## References

[1] Rachner TD Khosla S Hofbauer LC. Osteoporosis: now and the future. Lancet (2011) 377(9773):1276–87. doi: 10.1016/S0140-6736(10)62349-5 PMC3555696 21450337

[2] Bouxsein ML Boyd SK Christiansen BA Guldberg RE Jepsen KJ Muller R. Guidelines for assessment of bone microstructure in rodents using micro-computed tomography. J Bone Miner Res (2010) 25(7):1468–86. doi: 10.1002/jbmr.141 20533309

[3] Ulrich D Van Rietbergen B Laib A Ruegsegger P. The ability of three-dimensional structural indices to reflect mechanical aspects of trabecular bone. Bone (1999) 25(1):55–60. doi: 10.1016/s8756-3282(99)00098-8 10423022

[4] Maquer G Musy SN Wandel J Gross T Zysset PK. Bone volume fraction and fabric anisotropy are better determinants of trabecular bone stiffness than other morphological variables. J Bone Miner Res (2015) 30(6):1000–8. doi: 10.1002/jbmr.2437 25529534

[5] Alomari AH Wille ML Langton CM. Bone volume fraction and structural parameters for estimation of mechanical stiffness and failure load of human cancellous bone samples; in-vitro comparison of ultrasound transit time spectroscopy and X-ray muCT. Bone (2018) 107:145–53. doi: 10.1016/j.bone.2017.11.021 29198979

[6] Feng C Yao J Wang L Zhang X Fan Y. Idealized conductance: a new method to evaluate stiffness of trabecular bone. Int J Numer Method BioMed Eng (2021) 37(3):e3425. doi: 10.1002/cnm.3425 33289331

[7] Gibson LJ. Biomechanics of cellular solids. J Biomech (2005) 38(3):377–99. doi: 10.1016/j.jbiomech.2004.09.027 15652536

[8] Un K Bevill G Keaveny TM. The effects of side-artifacts on the elastic modulus of trabecular bone. J Biomech (2006) 39(11):1955–63. doi: 10.1016/j.jbiomech.2006.05.012 16824533

[9] Bevill G Easley SK Keaveny TM. Side-artifact errors in yield strength and elastic modulus for human trabecular bone and their dependence on bone volume fraction and anatomic site. J Biomech (2007) 40(15):3381–8. doi: 10.1016/j.jbiomech.2007.05.008 PMC2099450 17659290

[10] Zysset PK Guo XE Hoffler CE Moore KE Goldstein SA. Elastic modulus and hardness of cortical and trabecular bone lamellae measured by nanoindentation in the human femur. J Biomech (1999) 32(10):1005–12. doi: 10.1016/s0021-9290(99)00111-6 10476838

[11] Jaasma MJ Bayraktar HH Niebur GL Keaveny TM. Biomechanical effects of intraspecimen variations in tissue modulus for trabecular bone. J Biomech (2002) 35(2):237–46. doi: 10.1016/s0021-9290(01)00193-2 11784542

[12] Routh RH Rumancik S Pathak RD Burshell AL Nauman EA. The relationship between bone mineral density and biomechanics in patients with osteoporosis and scoliosis. Osteoporos Int (2005) 16(12):1857–63. doi: 10.1007/s00198-005-1951-z 15999291

[13] Sprecher CM Schmidutz F Helfen T Richards RG Blauth M Milz S. Histomorphometric assessment of cancellous and cortical bone material distribution in the proximal humerus of normal and osteoporotic individuals: significantly reduced bone stock in the metaphyseal and subcapital regions of osteoporotic individuals. Med (Baltimore) (2015) 94(51):e2043. doi: 10.1097/MD.0000000000002043 PMC4697966 26705200

[14] Silva MJ Gibson LJ. Modeling the mechanical behavior of vertebral trabecular bone: effects of age-related changes in microstructure. Bone (1997) 21(2):191–9. doi: 10.1016/s8756-3282(97)00100-2 9267695

[15] Silva MJ Keaveny TM Hayes WC. Computed tomography-based finite element analysis predicts failure loads and fracture patterns for vertebral sections. J Orthop Res (1998) 16(3):300–8. doi: 10.1002/jor.1100160305 9671924

[16] Yeh OC Keaveny TM. Biomechanical effects of intraspecimen variations in trabecular architecture: a three-dimensional finite element study. Bone (1999) 25(2):223–8. doi: 10.1016/s8756-3282(99)00092-7 10456389

[17] Yeh OC Keaveny TM. Relative roles of microdamage and microfracture in the mechanical behavior of trabecular bone. J Orthop Res (2001) 19(6):1001–7. doi: 10.1016/S0736-0266(01)00053-5 11780997

[18] Liu P Liang X Li Z Zhu X Zhang Z Cai L. Decoupled effects of bone mass, microarchitecture and tissue property on the mechanical deterioration of osteoporotic bones. Composites Part B: Engineering (2019) 177(11):1–8. doi: 10.1016/j.compositesb.2019.107436

[19] Du J Li S Silberschmidt VV. Remodelling of trabecular bone in human distal tibia: a model based on an in-vivo HR-pQCT study. J Mech Behav BioMed Mater (2021) 119. doi: 10.1016/j.jmbbm.2021.104506 33865068

[20] Barak MM Black MA. A novel use of 3D printing model demonstrates the effects of deteriorated trabecular bone structure on bone stiffness and strength. J Mech Behav BioMed Mater (2018) 78(2):455–64. doi: 10.1016/j.jmbbm.2017.12.010 PMC5758409 29241149

[21] Kuhn V Ivanovic N Recheis W. High resolution 3D-printing of trabecular bone based on micro-CT data. J Orthop Translat (2014) 4(2):238.

[22] Woo DG Kim CH Kim HS Lim D. An experimental-numerical methodology for a rapid prototyped application combined with finite element models in vertebral trabecular bone. Exp Mech (2008) 48(5):657–64. doi: 10.1007/s11340-007-9108-y

[23] Hokeun Kim YZ Zhao L. Process-level modeling and simulation for HP's multi jet fusion 3D printing technology. In: 2016 1st international workshop on cyber-physical production systems (CPPS) (Vienna, Austria: Institute of Electrical and Electronics Engineers (IEEE)) (2016).

[24] Singh AP Pervaiz S. Current status and prospects of multi-jet fusion (MJF) based 3D printing technology, in: ASME 2021 International Mechanical Engineering Congress and Exposition, Virtual, Online: American Society of Mechanical Engineers (ASME) (2021).

[25] Dai S Deng ZC Yu YJ Zhang K Wang SH Ye J. Orthotropic elastic behaviors and yield strength of fused deposition modeling materials: theory and experiments. Polymer Testing (2020) 87. doi: 10.1016/j.polymertesting.2020.106520

[26] Cory E Nazarian A Entezari V Vartanians V Muller R Snyder BD. Compressive axial mechanical properties of rat bone as functions of bone volume fraction, apparent density and micro-ct based mineral density. J Biomech (2010) 43(5):953–60. doi: 10.1016/j.jbiomech.2009.10.047 PMC4405886 20003979

[27] Waldorff EI Goldstein SA Mccreadie BR. Age-dependent microdamage removal following mechanically induced microdamage in trabecular bone in vivo. Bone (2007) 40(2):425–32. doi: 10.1016/j.bone.2006.08.011 17055351

[28] Yang H Butz KD Duffy D Niebur GL Nauman EA Main RP. Characterization of cancellous and cortical bone strain in the in vivo mouse tibial loading model using microCT-based finite element analysis. Bone (2014) 66:131–9. doi: 10.1016/j.bone.2014.05.019 24925445

[29] Yang H Xu X Bullock W Main RP. Adaptive changes in micromechanical environments of cancellous and cortical bone in response to in vivo loading and disuse. J Biomech (2019) 89:85–94. doi: 10.1016/j.jbiomech.2019.04.021 31047696

[30] Morgan EF Keaveny TM. Dependence of yield strain of human trabecular bone on anatomic site. J Biomech (2001) 34(5):569–77. doi: 10.1016/s0021-9290(01)00011-2 11311697

[31] Muller R Ruegsegger P. Analysis of mechanical properties of cancellous bone under conditions of simulated bone atrophy. J Biomech (1996) 29(8):1053–60. doi: 10.1016/0021-9290(96)00006-1 8817372

[32] Van Der Linden JC Homminga J Verhaar JA Weinans H. Mechanical consequences of bone loss in cancellous bone. J Bone Miner Res (2001) 16(3):457–65. doi: 10.1359/jbmr.2001.16.3.457 11277263

[33] Liu XS Sajda P Saha PK Wehrli FW Guo XE. Quantification of the roles of trabecular microarchitecture and trabecular type in determining the elastic modulus of human trabecular bone. J Bone Miner Res (2006) 21(10):1608–17. doi: 10.1359/jbmr.060716 PMC3225012 16995816

[34] Zheng L Huang X Li C Li P Lin Z Huang S. 3D printed trabeculae conditionally reproduce the mechanical properties of the actual trabeculae - a preliminary study. Heliyon (2022) 8(12):e12101. doi: 10.1016/j.heliyon.2022.e12101 36544825 PMC9761705

[35] Morgan EF Bayraktar HH Keaveny TM. Trabecular bone modulus-density relationships depend on anatomic site. J Biomech (2003) 36(7):897–904. doi: 10.1016/s0021-9290(03)00071-x 12757797

[36] Wald MJ Magland JF Rajapakse CS Bhagat YA Wehrli FW. Predicting trabecular bone elastic properties from measures of bone volume fraction and fabric on the basis of micromagnetic resonance images. Magn Reson Med (2012) 68(2):463–73. doi: 10.1002/mrm.23253 PMC3374911 22162036

[37] Keller TS. Predicting the compressive mechanical behavior of bone. J Biomech (1994) 27(9):1159–68. doi: 10.1016/0021-9290(94)90056-6 7929465

[38] Yang G Kabel J Van Rietbergen B Odgaard A Huiskes R Cowin SC. The anisotropic hooke's law for cancellous bone and wood. J Elast (1998) 53(2):125–46. doi: 10.1023/a:1007575322693 11543211

[39] Gibson LJ Ashby MF. Cellular solids : structure and properties. Cambridge, New York: Cambridge University Press (1997).

[40] Langton CM Whitehead MA Langton DK Langley G. Development of a cancellous bone structural model by stereolithography for ultrasound characterisation of the calcaneus. Med Eng Phys (1997) 19(7):599–604. doi: 10.1016/s1350-4533(97)00027-1 9457693

[41] Tellis BC Szivek JA Bliss CL Margolis DS Vaidyanathan RK Calvert P. Trabecular scaffolds created using micro CT guided fused deposition modeling. Mater Sci Eng C (2008) 28(1):171–8. doi: 10.1016/j.msec.2006.11.010 PMC3065838 21461176

[42] Zhang C Zhang L Liu L Lv L Gao L Liu N. Mechanical behavior of a titanium alloy scaffold mimicking trabecular structure. J Orthop Surg Res (2020) 15(1):40. doi: 10.1186/s13018-019-1489-y 32028970 PMC7006186

[43] Grzeszczak A Lewin S Eriksson O Kreuger J Persson C. The potential of stereolithography for 3D printing of synthetic trabecular bone structures. Materials (Basel) (2021) 14(13). doi: 10.3390/ma14133712 PMC8269906 34279283

[44] Yoon YJ Moon SK Hwang J. 3D printing as an efficient way for comparative study of biomimetic structures - trabecular bone and honeycomb. J Mechanical Sci Technology (2014) 28(11):4635–40. doi: 10.1007/s12206-014-1031-4

[45] Wood Z Lynn L Nguyen JT Black MA Patel M Barak MM. Are we crying Wolff? 3D printed replicas of trabecular bone structure demonstrate higher stiffness and strength during off-axis loading. Bone (2019) 127(8):635–45. doi: 10.1016/j.bone.2019.08.002 PMC6939675 31390534

[46] Dobson CA Sisias G Phillips R Fagan MJ Langton CM. Three dimensional stereolithography models of cancellous bone structures from mu CT data: testing and validation of finite element results. Proc Inst Mech Eng Part H (2006) 220(H3):481–4. doi: 10.1243/09544119H00405 16808081

[47] Hu J Wang JH Wang R Yu XB Liu Y Baur DA. Analysis of biomechanical behavior of 3D printed mandibular graft with porous scaffold structure designed by topological optimization. 3D Print Med (2019) 5(1):5. doi: 10.1186/s41205-019-0042-2 30874929 PMC6743138

[48] Zheng L Dai Y Zheng Y He X Wu M Zheng D. Medial tibial plateau sustaining higher physiological stress than the lateral plateau: based on 3D printing and finite element method. Biomed Eng Online (2022) 21(1):68. doi: 10.1186/s12938-022-01039-x 36114576 PMC9482229

[49] Thomsen JS Niklassen AS Ebbesen EN Bruel A. Age-related changes of vertical and horizontal lumbar vertebral trabecular 3D bone microstructure is different in women and men. Bone (2013) 57(1):47–55. doi: 10.1016/j.bone.2013.07.025 23899636

[50] Greenwood C Clement J Dicken A Evans P Lyburn I Martin RM. Age-related changes in femoral head trabecular microarchitecture. Aging Dis (2018) 9(6):976–87. doi: 10.14336/AD.2018.0124 PMC6284768 30574411

