# Supplementary material for: Studying trabecular bone samples demonstrates a power law relation between deteriorated structure and mechanical properties - a study combining 3D printing with the finite element method
Source: Front Endocrinol (Lausanne). 2023 Jun 2;14:1061758. doi: 10.3389/fendo.2023.1061758 (PMC10273262; doi:10.3389/fendo.2023.1061758)
Supplement: Supplementary file 1 [file DataSheet_1.docx]

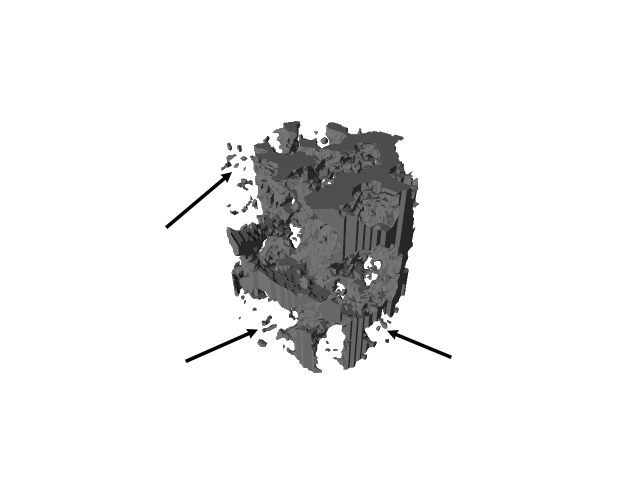


Supplementary Figure S1: The raw segmentation derived from CTAn software were noisy: full of spikes and unconnected elements as black arrows indicated.


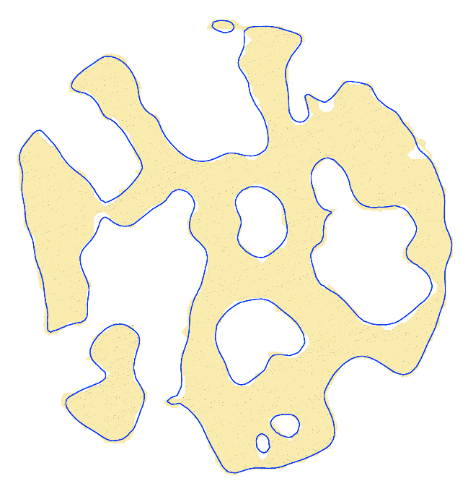


Supplementary Figure S2: Comparison between raw image and polished image in a random slices. The orange fill represent the raw image and blue line represent polished image. Took N2 sample at threshold of 65 for example.


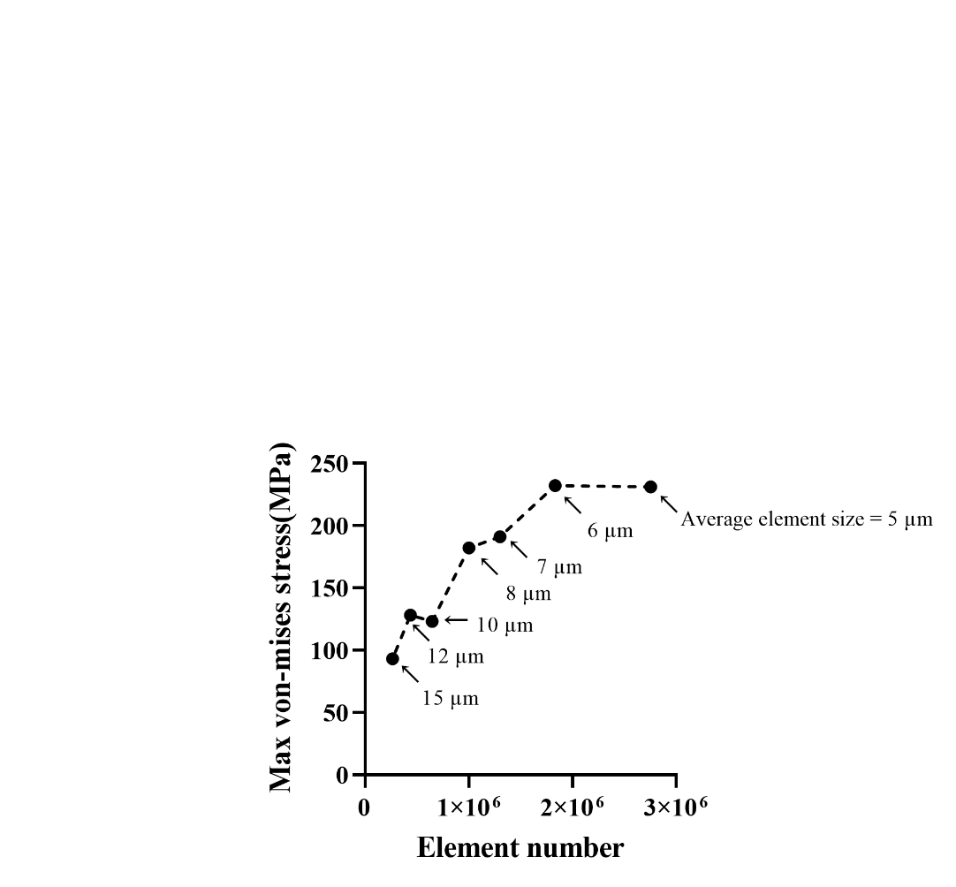


Supplementary Figure S3: Sensitivity analysis suggest that average element size of 8um is sufficient to converge (took OVX3 for clarity).

Supplementary Table S1. BV/TV of each sample at different global threshold and their side-artifact correction factor.

| Threshold | N1 | | N2 | | N3 | | OVX1 | | OVX2 | | OVX3 | |
| --- | --- | --- | --- | --- | --- | --- | --- | --- | --- | --- | --- | --- |
|  | BV/TV | Correction factor | BV/TV | Correction factor | BV/TV | Correction factor | BV/TV | Correction factor | BV/TV | Correction factor | BV/TV | Correction factor |
| 65 | 0.515 | 1.214 | 0.534 | 1.210 | 0.539 | 1.202 | 0.311 | 1.347 | 0.289 | 1.504 | 0.333 | 1.342 |
| 70 | 0.470 | 1.222 | 0.501 | 1.218 | 0.500 | 1.210 | 0.288 | 1.357 | 0.267 | 1.515 | 0.312 | 1.352 |
| 75 | 0.431 | 1.230 | 0.473 | 1.226 | 0.467 | 1.219 | 0.269 | 1.367 | 0.249 | 1.527 | 0.294 | 1.362 |
| 80 | 0.398 | 1.239 | 0.449 | 1.234 | 0.438 | 1.227 | 0.254 | 1.376 | 0.234 | 1.539 | 0.279 | 1.372 |
| 85 | 0.367 | 1.247 | 0.428 | 1.243 | 0.413 | 1.235 | 0.240 | 1.386 | 0.220 | 1.550 | 0.265 | 1.381 |
| 90 | 0.338 | 1.256 | 0.407 | 1.252 | 0.389 | 1.244 | 0.228 | 1.397 | 0.208 | 1.562 | 0.252 | 1.391 |
| 95 | 0.312 | 1.265 | 0.388 | 1.260 | 0.367 | 1.252 | 0.217 | 1.407 | 0.196 | 1.574 | 0.239 | 1.402 |
| 100 | 0.286 | 1.273 | 0.369 | 1.269 | 0.345 | 1.261 | 0.206 | 1.417 | 0.184 | 1.587 | 0.227 | 1.412 |
